# Rice yield penalty and quality deterioration is associated with failure of nitrogen uptake from regreening to panicle initiation stage under salinity

**DOI:** 10.3389/fpls.2023.1120755

**Published:** 2023-03-21

**Authors:** Yusheng Li, Zhiyong Ai, Yixue Mu, Tingcheng Zhao, Yicheng Zhang, Lin Li, Zheng Huang, Lixiao Nie, Mohammad Nauman Khan

**Affiliations:** ^1^ Sanya Nanfan Research Institute of Hainan University, Hainan University, Sanya, China; ^2^ College of Tropical Crops, Hainan University, Haikou, China; ^3^ National Innovation Center of Saline−Alkali Tolerant Rice in Sanya, Sanya, China; ^4^ Hunan Hybrid Rice Research Center, Changsha, China

**Keywords:** rice, salinity sensitivity, irrigating brine stage, grain yield, grain quality, N uptake

## Abstract

In recent years, the development and utilization of saline land for rice cultivation have effectively expanded grain productivity. Rice is a salt-sensitive crop, and the increasing salinity problem threatens rice yield and quality. Therefore, we conducted open field experiments to study the effect of salinity on different growth stages of rice. Irrigating saline treatment was conducted at three different growth stages: irrigating saline from the regreening stage to the panicle initiation stage (S1), irrigating saline from the panicle initiation stage to the flowering stage (S2), and irrigating saline from the flowering stage to the maturity stage (S3). Each treatment period lasted for about 30 days. At the same time, irrigating saline water from the regreening stage to the maturity stage (S4) treatment was added in 2022 to explore the performance of salt stress during the whole growth period of rice. Based on the treatment of these different saline irrigation growth periods, three saline concentrations were incorporated, including salinity 0‰ (T1), 3‰ (T2), and 6‰ (T3) concentrations. No irrigating saline during the whole growth period was also used as a control (CK). The results indicated that rice grain yield and quality were most sensitive to saline treatment during S1 among the three stress periods. At the S1 stage, salinity mainly reduced the nitrogen uptake, resulting in stunted plant growth, reducing tillering, yield, and yield components, and deteriorating the rice quality. Compared to the control, IE_N_ (grain yield over the total amount of N uptake in plants at maturity) was more sensitive at the S1 stage than S2 and S3 stages under salinity. Furthermore, the findings of our study suggest that under salinity, rice growth is not only directly affected by the higher sodium (Na^+^) content in plants, but the higher concentration of Na^+^ reduced the ability of plants to uptake nitrogen. Thus, more attention should be paid to the field management of the S1 stage, the most sensitive stage during rice cultivation in salinized areas. It is necessary to avoid salt damage to rice during this period and ensure irrigation with precious freshwater resources.

## 1 Introduction

Rice (*Oryza sativa* L.) has been cultivated as a staple food crop for 11,500 years, feeding about 56% of the world’s population and occupying an important position in food production (Chauhan, 2012; Verma et al., 2021). It is predicted that the world population will continue to grow to 11.2 billion by 2100 (United Nations, 2017). In comparison, Asia, Africa, and America, which have the fastest population growth as the major regions for rice consumption and production, will have a more rapid growth in future demand for rice than for other crops. Thus, there is an urgent need to increase rice production to meet the growing population’s food consumption demand and ensure global food security.

Soil salinization is getting a severe environmental issue worldwide, with approximately 1 billion hectares of global land affected by salinity, including more than 20% of irrigated farmland (Ivushkin et al., 2019; Negacz et al., 2022). In recent years, with rapid industrialization and urban expansion, the problem of global warming has gradually intensified, and meteorological disasters have occurred more frequently (Zhu et al., 2021). Increasing surface temperatures, prolonged droughts, and changes in rainfall patterns have caused increased surface evaporation, raising the accumulation of soil salts. Sea level rise due to glacial snow melt and ocean thermal expansion, and seawater intrusion further exacerbate coastal land salinization (Tzemi et al., 2020; Eswar et al., 2021; Wang et al., 2022). The increasing area of saline soils has become a major global problem limiting agricultural productivity and sustainable development, threatening food security. Rice, a moderately salt-sensitive crop (Sujatha et al., 2010), is negatively affected by salinity during growth and development, yield, and quality formation. It has been reported that 30 mmol L^-1^ NaCl (conductivity ~3 ds m^-1^) is considered low salt stress and has significantly hampered the growth and yield of rice plants, reducing yields by about one-third (Lutts et al., 1995; Thitisaksakul et al., 2015).

Rice has a complex response to salt stress and may use different mechanisms to cope with salt stress at different developmental stages. Previous studies (Zeng et al., 2001) reported that rice is more sensitive to salt stress at early growth stages (trifoliate stage-panicle initiation stage) than other reproductive stages and has the lowest yield at harvest. The analysis revealed that rice biomass and the number of tillers per plant were significantly reduced under early salt stress. The grain number and grain weight per panicle were significantly decreased under salt stress from the three-leaf stage to the panicle initiation stage and from the panicle initiation stage to the booting stage. In contrast, salt stress after the booting stage had no significant effect on rice yield. This is consistent with the finding by Asch et al. (1997) that salt stress reduces biomass and leaf area, thereby affecting the source-sink relationship and leading to a decrease in the reservoir (spikelet abortion and grain weight). In addition, Ebrahimi et al. (2012) reported that spike length, spike solid number, and spike weight were significantly reduced by salinity stress at early growth stages (tillering and panicle initiation stage) compared to late growth stages (tasseling and maturity stage) and irrigation with saline water at later stages of rice production had less adverse effects on rice growth. However, it has also been shown that the rice reproductive stage, the gestation stage, is the most sensitive to salt stress. It was found that salt stress at this stage had the most significant effect on economic traits such as plant height, the effective number of spikes, number of solid grains, set percentage, thousand-grain weight, and yield of rice, followed by the booting and tasseling stages (Zhu and Fan, 2021).

In summary, based on the findings of previous research work, it is not very clear which growth period rice is most sensitive to salt stress, and the related mechanisms are still largely unknown. Previous studies have been conducted mainly under potted conditions. Still, fewer studies have investigated rice sensitivity to salt stress at different reproductive stages under open field conditions, taking the entire reproductive period of rice as the starting point. Therefore, the present study was designed with the objectives (1) to comprehensively investigate the effects of salt stress on rice yield, rice quality, and nitrogen utilization at different growth periods under different salt concentrations; and (2) to elucidate the key periods of rice sensitivity to salt stress throughout the growth periods under field conditions.

## 2 Materials and methods

### 2.1 Experimental site

The experiments were conducted in Dadan Village, Yacheng Town, Yazhou District, Sanya City, Hainan Province, China (18°36’N, 109°15’E). The experimental base was located at the seashore with a complete salinity control and adjustable brine distribution system, which could simulate salt stress conditions realistically. The system includes a sequential connected water diversion system, brine distribution pool, and field irrigation system, which could directly pump seawater and underground freshwater in the brine pool to mix the required concentration of brine into the experimental field using pipeline facilities. The precipitation, total solar radiation, and average temperatures showed no obvious differences between the rice growing seasons. The precipitation in 2021 was 236.9 mm, 22.5% lower than that in 2022 (290.3 mm). The total solar radiation in 2021 was 2371.7 MJ m^-2^, 17.0% higher than that in 2022 (2026.4 MJ m^-2^). The maximum temperature, minimum temperature, and average temperature in 2021 and 2022 were 28.2°C, 23.8°C, 25.20°C, 26.2°C, 21.8°C, and 24.72°C, respectively. Before the experiment, single-season rice was planted in the paddy field for many years. The soil backgrounds of the two-year rice fields were similar. The pH, total nitrogen (N), available phosphorus, potassium, and organic matter in the upper 20 cm of the soil before rice planting was 6.82, 0.390 g kg^-1^, 13.13 mg kg^-1^, 226.72 mg kg^-1^, and 0.70%, respectively.

### 2.2 Experimental design

The proposed study was laid out in a split-split plot arrangement with four replications, and the area of each plot was 11.52 m^2^ (3.2 × 3.6 m). The irrigation brine period was allotted to main plot: only irrigating brine from the regreening stage to the panicle initiation stage (S1), only irrigating brine from the panicle initiation stage to the flowering stage (S2), only irrigating brine from the flowering stage to the maturity stage (S3), and irrigating brine from the regreening stage to the maturity stage (S4). At the same time, S4 was a supplemental trial added in 2022 compared with 2021 to explore the performance of rice under salt stress during the whole growth period. The irrigation saline concentration was allotted to the subplot, including salt concentration 0‰ (T1), salt concentration 3‰ (T2), and salt concentration 6‰ (T3). Two hybrid rice varieties, Chaoyouqianhao (CY1000) and Longliangyou506 (LLY506), were allotted to sub-sub plots, which showed similar performance in plant type, growth period, disease resistance, and lodging resistance. CY1000 was bred by Hunan Nianfeng Seed Industry Technology Co., LTD and Hunan Hybrid Rice Research Center, while Hainan University bred LLY506. The start irrigating brine time of S1 and S4 was 12 days after transplanting (DAT). However, the start irrigating brine times of S2 and S3 were different among different varieties and years. The start irrigating brine time of S2 for CY1000 and LLY506 was at 44 DAT and 45 DAT in 2021 and 42 DAT and 43 DAT in 2022. While the start irrigating brine time of S3 for CY1000 and LLY506 was at 72 DAT and 77 DAT in 2021 and 70 DAT and 75 DAT in 2022. The duration of each irrigation brine represented by S1, S2 and S3 are approximately 30 days. At the same time, all other stages were irrigated with fresh water without added salts except for the set 30-day saline stress time. At the end of one period, the saline areas were rinsed with fresh water several times to restore the salt in the areas to the state of no saline irrigation.

Pre-germinated seeds were sown in nurseries on 20 January 2021 and 13 January 2022. The transplanting dates were 15 February 2021, and 5 February 2022. All plots were plowed and puddled before transplanting. Seedlings were transplanted into the paddy soil with a hill spacing of 20 × 20 cm, with 2 seedlings per hill. Plants were fertilized with 150:60:100 kg ha^-1^ of N: P: K. The nitrogen fertilizer was applied as basal fertilizer, tillering fertilizer, and booting fertilizer at the ratio of 1:1:1. Phosphorus fertilizer was applied once as basal fertilizer. Potassium fertilizer was applied as basal fertilizer and booting fertilizer at the ratio of 1:1. The sources of N, P, and K fertilizers were urea (46.4% N), calcium superphosphate (16.0% P_2_O_5_) and potassium chloride (60.0% K_2_O), respectively. According to the conversion of each plot area (11.52 m^2^), each independent plot needed about 376g urea, 432g calcium superphosphate and 192g potassium chloride and the fertilizers were applied artificially and evenly. According to the early warning of disease and insect disaster issued by the local agricultural technology extension service, combined with field observation, we regularly sprayed with pesticides from the middle tillering period of rice, and periodically changed the pharmaceutical brand. At the same time, measures such as hanging bird-repellent nets, surrounding rodent-repellent mulching film, spraying rodent poison and bird repellent agent were taken to prevent yield loss during rice flowering period.

### 2.3 Data recorded

#### 2.3.1 Tiller or panicle number, plant height and total dry matter weight (TDW)

At the panicle initiation stage (PI), flowering stage (FL), and maturity stage (MS), 6 plants (0.24 m^2^) were randomly taken for growth analysis in each plot. The plants were washed, and the tiller number and plant height were recorded. After removing the roots with scissors, the plants were divided into straw and panicles. The samples were dried in an oven at 105°C for half an hour and then at 80°C for 2 days until they reached constant weight for weighing the TDW.

#### 2.3.2 Grain yield and its components

In the maturity stage of rice, grain yield was determined from a 3 m^2^ area in the middle of each plot, wind-selected to remove impurities, weighed for total filled spikelets, and adjusted to the standard moisture content of 0.14 g H_2_O g^-1^ fresh weight. After removed the positions of the side of the plots, six plants (0.24 m^2^) with similar growth in the middle of each plot were randomly sampled for data regarding rice yield components at MS. The panicle number was counted in each sample to determine the panicle number per m^2^, and then the plants were separated into straw and panicles. Mixed rachises in the spikelets were selected through hand-threshing. All spikelets were submerged in tap water to separate the filled grains from the others (half-filled spikelets and unfilled spikelets). After air drying, the unfilled spikelets were separated from the half-filled spikelets by winnowing. Then, filled spikelets, half-filled spikelets, and unfilled spikelets were weighed. Three subsamples consisting of 30.0 g of filled spikelets, 2.0 g of unfilled spikelets, and all half-filled spikelets were used to count the number of filled, unfilled, and half-filled spikelets, respectively. After oven-drying at 80 °C to a constant weight, the dry weights of the straw and filled, half-filled and unfilled spikelets were determined. Therefore, the number of panicles per unit area, the number of spikelets per panicle, grain-filling percentage, 1000-grain weight, and harvest index were calculated.

#### 2.3.3 Determination of nitrogen content and nitrogen utilization

The samples of straw and grains that had been dried to constant weight, as described in section 2.3.1 were crushed and ground using a hybrid ball mill at a frequency of 30.0 s^−1^ for 1 min (MM400, RETSCH, Germany). The balls were cleaned with distilled water and dried among the interval milling to avoid cross contamination. Afterward, 0.1 g of ground sample from each treatment was digested using the H_2_SO_4_-H_2_O_2_ digestion method, and N content of samples was determined by an Automatic discontinuous chemical analyzer (Clever Chem Anna, Dechem-Tech, Germany) to calculate total nitrogen accumulation at different periods and the internal N use efficiency (IE_N_, grain yield over the total amount of N uptake in plants at maturity).

#### 2.3.4 Determination of grain quality

Before grain quality evaluation, the samples were air dried to 12–13% moisture content and stored for over three months. Two subsamples with 30 g grains were dehusked with a laboratory husker machine (NA.JLG-2058, Taizhou Luqiao Nongao grain equipment factory, China) to produce brown rice (whole caryopsis with intact pericarp and embryo). The resultant brown rice from each subsample was milled for 30 s with a laboratory milling machine (JA-NMJ, Taizhou Luqiao Jingao grain equipment factory, China) to yield milled rice. Finally, the milled rice from each subsample was classified into head rice (≥ 4/5 whole grain length) and broken rice with a laboratory separator machine (FOS-130, China). The weights of brown rice, milled rice, and head rice of each subsample were measured to determine brown rice percentage (BRP), milled rice percentage (MRP), and head rice percentage (HRP) with the following formulas: BRP = weight of brown rice/weight of subsample × 100%, MRP = weight of milled rice/weight of subsample × 100%, HRP = weight of head rice/weight of subsample × 100%. The appearance quality characteristics, including chalky kernel percentage (CKP) and chalkiness degree (CD) of the head rice samples, were analyzed with a rice quality analyzer (SC-E, Hangzhou Wanshen Test Technology Corporation, China). About 50 g milled rice was weighed for each treatment, and STM module group of near-infrared grain quality analyzer (Infratec 1241 Grain Analyzer, Foss Tecator, Sweden) was selected, and the pathlength selected as 18mm. The ANN global scaling model “MR173242” was used to determine the nutrition and taste quality characteristics, including protein content, amylose content (AC), and taste value.

#### 2.3.5 Determination of Na^+^ and K^+^ in rice aboveground plant

At the panicle initiation stage (PI), flowering stage (FL), and maturity stage (MS), 3 plants were randomly taken for subsequent determination in each plot. After washing and removing the roots, the aboveground samples were dried to constant weight in an oven and fully ground with a hybrid ball mill. About 0.1 g of crushed sample from each treatment was digested with HNO_3_ and HClO_4_ (4:1, v/v) concentrated in a microwave oven (Mars, CEM Inc, New York, USA). The concentration of Na^+^ and K^+^ were determined by a flame photometer (M410, Sherwood Scientific, Cambridge, UK).

#### 2.3.6 Data analysis

Data were analyzed through analysis of variance (ANOVA) using Statistix 9.0 software (Analytical Software, Tallahassee, FL, USA). The differences between treatments were separated using the least significance difference (LSD) test at the 0.05, 0.01, and 0.001 probability levels Sigmaplot 14.0 was used for the graphical presentation of the data.

## 3 Results

### 3.1 Effect of irrigating brine at different growth stages with various saline concentrations on plant height, tiller number, and TDW of rice

**Figure 1:**
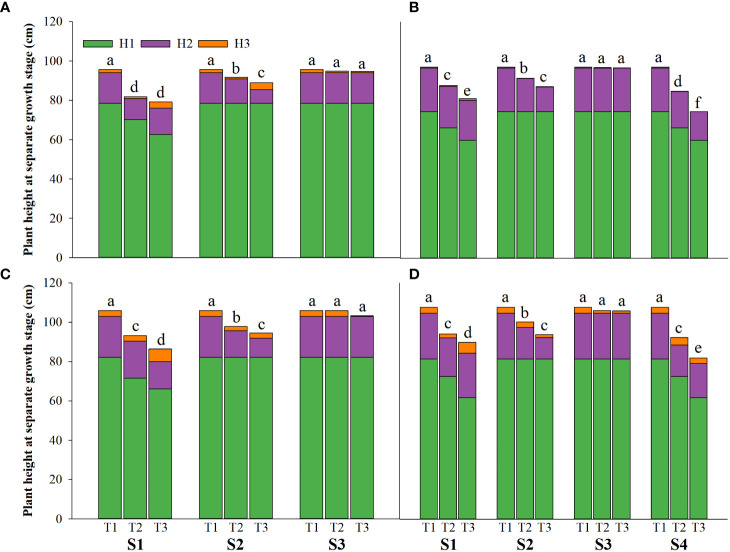
Effects of irrigating brine at different growth stages with different brine concentrations on the plant height of CY1000 **(A, B)**, LLY506 **(C, D)** in 2021 **(A, C)** and in 2022 **(B, D)** H1 represents plant height at panicle initiation stage; H2 represents plant height at the flowering stage; H3 represents plant height at maturity stage. S1 represents irrigating saline from the regreening stage to the panicle initiation stage; S2 represents irrigating saline from the panicle initiation stage to the flowering stage; S3 represents irrigating saline from the flowering stage to the maturity stage; S4 represents irrigating saline from the regreening stage to the maturity stage. T1, T2 and T3 represent the 0‰ saline, 3‰ saline and 6‰ saline, respectively. Different lower-case letters represent a significant difference at the maturity stage at 0.05 level according to the LSD test.

**Figure 2:**
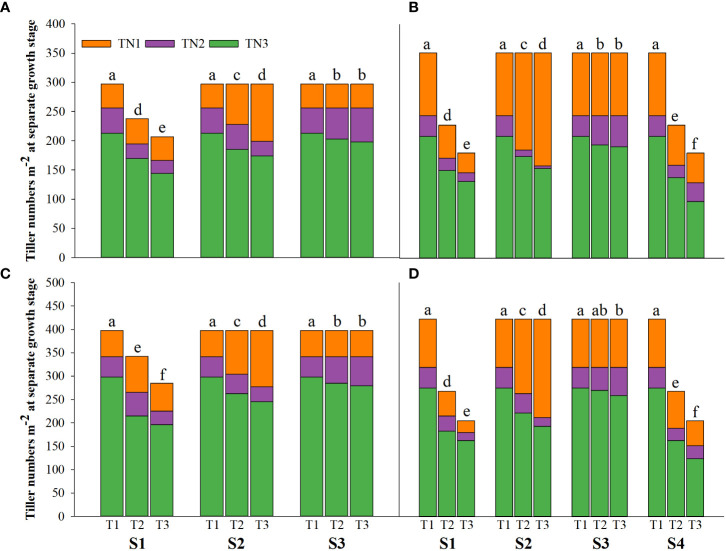
Effects of irrigating brine at different growth stages with different brine concentrations on tiller numbers of CY1000 **(A, B)**, LLY506 **(C, D)** in 2021 **(A, C)** and in 2022 **(B, D)**. TN1 represents tiller numbers at the panicle initiation stage; TN2 represents tiller numbers at the flowering stage; TN3 represents tiller numbers at the maturity stage. S1 represents irrigating saline from the regreening stage to the panicle initiation stage; S2 represents irrigating saline from the panicle initiation stage to the flowering stage; S3 represents irrigating saline from the flowering stage to the maturity stage; S4 represents irrigating saline from the regreening stage to the maturity stage. T1, T2 and T3 represent the 0‰ saline, 3‰ saline and 6‰ saline, respectively. Different lower-case letters represent a significant difference at the maturity stage at 0.05 level according to the LSD test.

**Figure 3:**
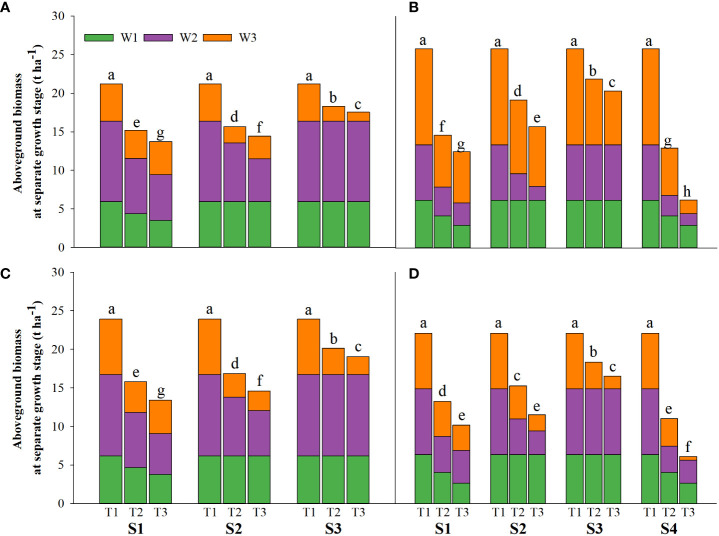
Effects of irrigating brine at different growth stages with different brine concentrations on aboveground biomass of CY1000 **(A, B)**, LLY506 **(C, D)** in 2021 **(A, C)** and in 2022 **(B, D)**. W1 represents aboveground biomass at the panicle initiation stage; W2 represents aboveground biomass at the flowering stage; W3 represents aboveground biomass at the maturity stage. S1 represents irrigating saline from the regreening stage to the panicle initiation stage; S2 represents irrigating saline from the panicle initiation stage to the flowering stage; S3 represents irrigating saline from the flowering stage to the maturity stage; S4 represents irrigating saline from the regreening stage to the maturity stage. T1, T2 and T3 represent the 0‰ saline, 3‰ saline and 6‰ saline, respectively. Different lower-case letters represent a significant difference at the maturity stage at 0.05 level according to the LSD test.

Salinity stress at different growth stages with various saline concentrations significantly affected the plant height, tiller number (panicle number), and TDW of rice (**Figures 1**–**3**). During S4, these three indexes of CY1000 and LLY506 decreased the most with increasing saline concentrations. On the other three same duration of salinization treatments (S1, S2 and S3), we could find the effect of salinity on the plant height, tiller number, and TDW of both rice varieties at different growth stages in the sequence of S1 > S2 > S3. When irrigating brine at S1, the plant height of CY1000 and LLY506 decreased by 14.4%−17.2%, 12.1%−18.6%, and 9.6%−16.6% and 12.5%−16.5% in 2021 and 2022, respectively; the tiller number of CY1000 and LLY506 decreased by 20.1%−32.3%, 28.0%−34.2%, and 28.0%−37.0% and 33.6%−40.9% in 2021 and 2022, respectively; the TDW of CY1000 and LLY506 decreased by 28.3%−35.3%, 33.9%−43.9%, and 43.4%−51.6% and 39.8%−53.8% in 2021 and 2022, respectively. In contrast, there were no significant differences in plant height for either variety between S3 and the treatment without irrigating brine from transplanting to harvesting (CK) at maturity. TDW decreased gradually when the saline concentration increased during S3. The tiller number also decreased during S3, but there was no significant difference between the two salinities (T2 and T3). In conclusion, under the same time of stress treatment (regardless of treatment S4), the irrigating brine stage with the most significant impact on rice plant height, tiller number (panicle number), and TDW were during S1 for both varieties in both growing seasons.

### 3.2 Effects of irrigating brine at different growth periods with various saline concentrations on grain yield and its components in rice

The yield loss of rice under salinity stress was the greatest at S4, while the yield components such as spikelets per m^2^, the number of spikelets per panicle, the filled grain rates, and 1000-grain weight were significantly reduced compared with CK, considering that treatment S4 lasted the most prolonged period. Compared to CK, the grain yield of CY1000 and LLY506 decreased by 14.3%−91.4% and 16.7%−90.6% due to salinity stress treatment across the two years, respectively. And the effect of salinity on the grain yield of both rice varieties at different growth stages was in the sequence of S4 > S1 > S2 > S3 (**Tables 1**, **2**). Among the three same duration of salinization treatments, irrigating brine at S1 and S2 significantly decreased the number of spikelets per panicle and spikelets per m^2^. No significant impact on spikelets per m^2^ was observed in either variety at S3. The spikelets per m^2^ of CY1000 and LLY506 decreased by 20.1%−32.3%, 28.0%−34.2%, and 28.0%−37.0% and 33.6%−40.9% in 2021 and 2022, respectively when the plants were irrigated brine at S1 (**Supplementary Tables 1, 2**). Meanwhile, the number of spikelets per panicle of CY1000 and LLY506 decreased by 29.4%−46.6%, 28.1%−39.5%, and 29.6%−43.1% and 22.5%−36.9% in 2021 and 2022, respectively. These resulted in decreases in the grain yield of CY1000 and LLY506 of 52.5%−77.2%, 57.0%−76.4%, and 53.5%−73.5% and 52.3%−66.7% in 2021 and 2022, respectively. The effect of irrigating brine at S1 on grain yield in 2021 was similar to 2022. Both rice varieties were most sensitive to irrigating brine treatment during S1 among the three same times of salinity stress treatments (regardless of treatment S4).

**Table 1:** Effects of irrigated saline water and different saline concentrations at different growth stages on rice yield and its constitutive factors (2021).

Variety	Irrigation period	Salinity	Panicles (no.m^-2^)	Spikelets panicle^-1^	Filled grains (%)	1000-grain weight (g)	Grain yield (t ha^-1^)
V1	S1	T1	212.5 a	203.5 a	84.9 a	24.5 a	9.13 a
		T2	169.8 d	143.6 e	81.9 b	20.7 e	4.34 f
		T3	143.8 e	108.7 f	75.5 d	19.3 f	2.08 g
	S2	T1	212.5 a	203.5 a	84.9 a	24.5 a	9.13 a
		T2	185.4 c	185.4 c	77.7 c	22.2 d	6.20 d
		T3	174.0 d	174.4 d	73.7 e	20.7 e	5.23 e
	S3	T1	212.5 a	203.5 a	84.9 a	24.5 a	9.13 a
		T2	203.1 b	195.5 b	78.4 c	23.4 b	7.81 b
		T3	197.9 b	187.9 c	74.4 de	22.6 c	6.80 c
V2	S1	T1	297.9 a	180.2 a	89.4 a	21.8 a	10.71 a
		T2	214.6 e	129.6 d	82.1 b	18.2 e	4.61 f
		T3	195.9 f	109.1 e	77.4 d	17.6 f	2.53 g
	S2	T1	297.9 a	180.2 a	89.4 a	21.8 a	10.71 a
		T2	262.5 c	156.4 c	79.7 c	19.0 d	7.16 d
		T3	244.8 d	134.8 d	74.9 e	18.2 e	5.21 e
	S3	T1	297.9 a	180.2 a	89.4 a	21.8 a	10.71 a
		T2	284.4 b	166.4 b	80.0 c	20.8 b	9.16 b
		T3	279.2 b	156.8 c	75.6 e	20.2 c	8.75 c
ANOVA		V	***	***	**	***	**
		S	***	***	***	***	***
		T	***	***	***	***	***
		V*S	***	***	ns	**	***
		V*T	***	ns	***	***	***
		S*T	***	***	***	***	***
		V*S*T	**	***	ns	**	***

S1 represents irrigating saline from the regreening stage to the panicle initiation stage; S2 represents irrigating saline from the panicle initiation stage to the flowering stage; S3 represents irrigating saline from the flowering stage to the maturity stage. T1, T2 and T3 represent the 0‰ saline, 3‰ saline and 6‰ saline, respectively. V1 and V2 represent the varieties of CY1000 and LLY506, respectively. Different lower-case letters of the same variety represent significant differences at 0.05 probability level according to LSD. *** represents the significant difference at the 0.001 level according to the LSD test, ** represents the significant difference at the 0.01 level according to the LSD test, * represents the significant difference at the 0.05 level according to the LSD test, and ns represents no significant difference.

**Table 2:** Effects of irrigated saline water and different saline concentrations at different growth stages on rice yield and its constitutive factor (2022).

Variety	Irrigation period	Salinity	Panicles (no.m^-2^)	Spikelets panicle^-1^	Filled grains (%)	1000-grain weight (g)	Grain yield (t ha^-1^)
V1	S1	T1	208.3 a	220.2 a	87.1 a	24.5 a	9.50 a
		T2	150.0 d	155.1 e	83.2 b	21.3 e	4.42 f
		T3	131.3 e	125.2 f	78.6 c	20.4 f	2.52 h
	S2	T1	208.3 a	220.2 a	87.1 a	24.5 a	9.50 a
		T2	174.0 c	189.4 cd	80.5 bc	22.8 c	6.36 d
		T3	153.1 d	179.6 d	74.4 d	21.8 d	4.94 e
	S3	T1	208.3 a	220.2 a	87.1 a	24.5 a	9.50 a
		T2	193.8 b	208.0 b	79.3 c	23.9 b	8.14 b
		T3	190.7 b	193.8 c	72.7 d	22.9 c	7.18 c
	S4	T1	208.3 a	220.2 a	87.1 a	24.5 a	9.50 a
		T2	137.5 e	154.9 e	75.1 d	20.8 f	3.75 g
		T3	96.9 f	86.65 g	59.0 e	18.4 g	0.82 i
V2	S1	T1	275.0 a	192.6 a	87.2 a	22.0 a	10.24 a
		T2	182.5 d	149.3 d	81.8 b	19.1 e	4.88 e
		T3	162.5 e	121.5 e	79.4 bc	17.8 g	3.41 f
	S2	T1	275.0 a	192.6 a	87.2 a	22.0 a	10.24 a
		T2	221.3 c	165.5 c	80.2 bc	20.1 d	6.09 d
		T3	192.5 d	146.5 d	71.3 d	19.2 e	4.68 e
	S3	T1	275.0 a	192.6 a	87.2 a	22.0 a	10.24 a
		T2	270.0 ab	176.7 b	78.0 c	21.2 b	8.53 b
		T3	258.8 b	165.6 c	73.6 d	20.6 c	7.73 c
	S4	T1	275.0 a	192.6 a	87.2 a	22.0 a	10.24 a
		T2	162.5 e	126.2 e	73.5 d	18.6 f	3.63 f
		T3	123.8 f	65.2 f	55.3 e	15.3 h	0.96 g
ANOVA		V	***	***	*	***	**
		S	***	***	***	***	***
		T	***	***	***	***	***
		V*S	***	**	ns	ns	***
		V*T	***	ns	ns	ns	***
		S*T	***	***	***	***	***
		V*S*T	**	*	ns	ns	ns

S1 represents irrigating saline from the regreening stage to the panicle initiation stage; S2 represents irrigating saline from the panicle initiation stage to the flowering stage; S3 represents irrigating saline from the flowering stage to the maturity stage; S4 represents irrigating saline from the regreening stage to the maturity stage. T1, T2 and T3 represent the 0‰ saline, 3‰ saline and 6‰ saline, respectively. V1 and V2 represent the varieties of CY1000 and LLY506, respectively. Different lower-case letters of the same variety represent significant differences at 0.05 probability level according to LSD. *** represents the significant difference at the 0.001 level according to the LSD test, ** represents the significant difference at the 0.01 level according to the LSD test, * represents the significant difference at the 0.05 level according to the LSD test, and ns represents no significant difference.

The grain yields of both rice varieties decreased as the saline concentrations increased for the four irrigation brine periods (**Tables 1**, **2**), while the grain yields were significantly different compared with CK. In addition, the grain yields of CY1000 and LLY506 decreased the most, by 24.4%−91.4% and 18.3%−90.6% under the T3 treatment, respectively. There were significant interactions between irrigating brine at different growth stages and saline concentrations on grain yield, spikelets per panicle, spikelets per m^2^, filled grain rate, and harvest index (P < 0.01).

### 3.3 Effects of irrigating brine at different growth periods with various saline concentrations on nitrogen content and nitrogen utilization of rice

Saline irrigation at different growth periods and concentrations significantly affected nitrogen accumulation and IE_N_ in rice at all fertility stages. As shown in **Tables 3**, **4**, salt stress significantly inhibited nitrogen accumulation at each fertility stage of rice, and nitrogen accumulation significantly decreased with the salt concentration increasing. Nitrogen uptake at all fertility stages showed a pattern of S3 > S2 > S1 > S4. Compared with the control, irrigation with saline of a concentration of 3‰ at the S3 period had less effect on IE_N_. Compared with the control, among the three treatments (S1, S2 and S3) with the same time salt stress, IE_N_ was the most sensitive under the S1 treatment, reducing IE_N_ by 20.6%−53.4%, 28.9%−50.8% and 18.2%−41.2%, 15.9%−23.4% in CY1000 and LLY506 in 2021 and 2022, respectively. The order of the effect of irrigated saline water on IE_N_ of CY1000 and LLY506 in different periods was S4 > S1 > S2 > S3, and the trend of reduction was significantly different among treatments (P < 0.05).

**Table 3:** Effects of irrigation brine and different brine concentrations at different growth stages on nitrogen uptake in rice (2021).

Variety	Irrigation period	Salinity	Aboveground nitrogen uptake at different growth stages (kg ha^-1^)				IE_N_ (kg kg^-1^)
			PI	FL	MA		
					Straw	Grain	
V1	S1	T1	96.17 a	178.13 a	114.66 a	112.01 a	40.30 a
		T2	51.11 b	108.15 cd	81.67 cd	55.15 e	31.98 c
		T3	32.50 c	85.39 d	74.07 d	37.71 f	18.77 d
	S2	T1	96.17 a	178.13 a	114.66 a	112.01 a	40.30 a
		T2	96.17 a	136.83 b	87.70 c	78.30 cd	37.62 ab
		T3	96.17 a	114.68 bc	83.03 cd	68.04 d	34.64 bc
	S3	T1	96.17 a	178.13 a	114.66 a	112.01 a	40.30 a
		T2	96.17 a	178.13 a	103.25 ab	93.94 b	39.76 a
		T3	96.17 a	178.13 a	100.63 b	80.56 c	37.72 ab
V2	S1	T1	102.93 a	183.40 a	118.72 a	115.54 a	45.93 a
		T2	62.44 b	123.24 c	85.19 cd	57.60 d	32.66 b
		T3	35.41 c	92.58 d	69.34 d	42.84 e	22.58 c
	S2	T1	102.93 a	183.40 a	118.72 a	115.54 a	45.93 a
		T2	102.93 a	148.30 b	94.36 bc	81.01 c	41.23 a
		T3	102.93 a	125.69 c	84.85 cd	66.08 d	35.08 b
	S3	T1	102.93 a	183.40 a	118.72 a	115.54 a	45.93 a
		T2	102.93 a	183.40 a	105.68 ab	101.45 b	44.26 a
		T3	102.93 a	183.40 a	107.18 ab	93.85 b	43.52 a
		V	*	ns	ns	**	*
ANOVA		S	***	***	***	***	***
		T	***	***	***	***	***
		V*S	ns	ns	ns	ns	ns
		V*T	ns	ns	ns	ns	ns
		S*T	***	***	*	***	***
		V*S*T	ns	ns	ns	ns	ns

S1 represents irrigating saline from the regreening stage to the panicle initiation stage; S2 represents irrigating saline from the panicle initiation stage to the flowering stage; S3 represents irrigating saline from the flowering stage to the maturity stage. T1, T2 and T3 represent the 0‰ saline, 3‰ saline and 6‰ saline, respectively. V1 and V2 represent the varieties of CY1000 and LLY506, respectively. Different lower-case letters of the same variety represent significant differences at 0.05 probability level according to LSD. *** represents the significant difference at the 0.001 level according to the LSD test, ** represents the significant difference at the 0.01 level according to the LSD test, * represents the significant difference at the 0.05 level according to the LSD test, and ns represents no significant difference.

**Table 4:** Effects of irrigation brine and different brine concentrations at different growth stages on nitrogen uptake in rice (2022).

Variety	Irrigation period	Salinity	Aboveground nitrogen uptake at different growth stages (kg ha^-1^)				IE_N_ (kg kg^-1^)
			PI	FL	MA		
					Straw	Grain	
V1	S1	T1	96.32 a	168.77 a	125.66 a	117.11 a	39.24 a
		T2	59.09 b	80.40 cd	76.80 de	62.51 c	32.10 c
		T3	34.25 c	68.24 d	66.87 ef	42.30 d	23.09 d
	S2	T1	96.32 a	168.77 a	125.66 a	117.11 a	39.24 a
		T2	96.32 a	118.36 b	99.95 bc	89.11 b	33.71 bc
		T3	96.32 a	100.19 bc	84.63 cd	68.54 c	32.34 c
	S3	T1	96.32 a	168.77 a	125.66 a	117.11 a	39.24 a
		T2	96.32 a	168.77 a	111.13 ab	109.35 a	37.09 a
		T3	96.32 a	168.77 a	103.54 b	90.81 b	36.93 ab
	S4	T1	96.32 a	168.77 a	125.66 a	117.11 a	39.24 a
		T2	59.09 b	74.34 d	61.79 f	58.96 c	31.21 c
		T3	34.25 c	39.39 e	34.89 g	18.17 e	15.42 e
V2	S1	T1	93.46 a	158.61 a	104.08 a	112.88 a	47.31 a
		T2	50.85 b	87.74 cd	67.12 def	55.98 e	39.79 bc
		T3	30.32 c	68.72 de	56.47 f	38.32 f	36.23 c
	S2	T1	93.46 a	158.61 a	104.08 a	112.88 a	47.31 a
		T2	50.85 b	113.22 b	72.09 de	73.09 d	42.36 ab
		T3	30.32 c	95.79 bc	57.80 ef	53.16 e	42.34 ab
	S3	T1	93.46 a	158.61 a	104.08 a	112.88 a	47.31 a
		T2	93.46 a	158.62 a	88.22 bc	95.32 b	46.62 a
		T3	93.46 a	158.63 a	81.28 cd	86.88 c	46.10 a
	S4	T1	93.46 a	158.63 a	104.08 a	112.88 a	47.31 a
		T2	50.85 b	71.12 cde	58.67 ef	40.86 f	36.92 bc
		T3	30.32 c	48.72 e	37.66 g	13.56 g	18.97 d
		V	ns	ns	**	**	**
ANOVA		S	***	***	***	***	***
		T	***	***	***	***	***
		V*S	ns	ns	**	ns	ns
		V*T	ns	ns	ns	*	ns
		S*T	***	***	***	***	***
		V*S*T	ns	ns	ns	ns	ns

S1 represents irrigating saline from the regreening stage to the panicle initiation stage; S2 represents irrigating saline from the panicle initiation stage to the flowering stage; S3 represents irrigating saline from the flowering stage to the maturity stage; S4 represents irrigating saline from the regreening stage to the maturity stage. T1, T2 and T3 represent the 0‰ saline, 3‰ saline and 6‰ saline, respectively. V1 and V2 represent the varieties of CY1000 and LLY506, respectively. Different lower-case letters of the same variety represent significant differences at 0.05 probability level according to LSD. *** represents the significant difference at the 0.001 level according to the LSD test, ** represents the significant difference at the 0.01 level according to the LSD test, * represents the significant difference at the 0.05 level according to the LSD test, and ns represents no significant difference.

### 3.4 Effects of irrigating brine at different growth periods with various saline concentrations on grain quality

Saline irrigation at different growth periods and salt concentrations significantly affected the processing quality, appearance quality, nutritional and palatability quality of rice seeds and deteriorated rice quality (**Tables 5**, **6**). In the S1 and S4 periods, brown rice and milled rice percentages in rice processing quality decreased significantly with the increase in salt concentration (**Supplementary Tables 1, 2**). At the same time, no significant impacts were observed in S2 and S3 stages compared to CK. S4 treatment was irrigated with saline water from regreening to maturity, resulting in the greatest decrease in whole refined rice percentage in CY1000 and LLY506 in 2022, 15.5%−31.0% and 16.5%−44.5%, respectively. Among the remaining three treatments of salt stress at different fertility stages, the whole concentrate rice percentage of two rice varieties was most sensitive to S1 irrigation brine treatment. The head rice rates of CY1000 and LLY506 were reduced by 6.1%−21.1%, 8.1%−15.0% and 11.3%−17.5%, and 10.6%−19.2% in 2021 and 2022, respectively. The order of effect of irrigation salinity on rice processing quality of CY1000 and LLY506 at different fertility stages was S4 > S1 > S2 > S3, with significant (P < 0.05) difference in reduction trend among treatments. The saline irrigation at different stages of rice fertility increased the chalky grain rate and chalkiness of rice appearance quality, and they both increased significantly with increasing salt concentration. The greatest increases in chalky grain rate and chalkiness were observed in CY1000 and LLY506 in S4 compared with CK, ranging from 102.3%−225.1%, 108.4%−263.2%, and 106.1%−168.6%, 89.5%−155.8%, respectively. Among the remaining three treatments of salt stress at different fertility stages, the chalk grain percentage and chalkiness of the two rice varieties were the most sensitive to S1 irrigation saline treatment. The chalky grain rate of CY1000 and LLY506 increased by 110.0%−169.4%, 133.3%−160.8%, and 75.2%−142.3%, 67.1%−107.1% under S1 irrigation saline treatment in 2021 and 2022, respectively. And chalkiness increased by 139.6%−278.4%, 103.9%−165.4% and 85.5%−158.0% and 69.8%−111.9%, respectively. The effects of irrigation saline at different fertility stages on chalkiness and chalkiness of CY1000 and LLY506 were in the same trend, with the order of effects being S4 > S1 > S2 > S3, and the increasing trend differed significantly among treatments (P < 0.05). The protein content of rice of both rice varieties irrigated with saline water at different fertility stages increased with increasing salt concentration at the same period, which was highly significantly affected by saline water concentration (P < 0.001). The protein content of CY1000 and LLY506 under saline water stress at the 3‰ concentration at the S3 stage was not significantly different compared with CK. The protein content of CY1000 and LLY506 was affected by irrigation saline at different fertility stages in the order of S4 > S1 > S2 > S3, while the amylose content and taste value of both varieties decreased under irrigation saline at different fertility stages in the opposite order of protein content, in the order of S4 > S1 > S2 > S3. The amylose content and taste value were significantly (P < 0.05) affected by the period of salt stress and salt concentration among treatments.

**Table 5:** Effect of irrigated saline water and different saline concentrations at different growth stages on grain quality (2021).

Variety	Irrigation period	Salinity	Head rice (%)	Chalky grain (%)	Chalkiness (%)	Protein content (%)	Amylose content (%)	Taste value
V1	S1	T1	63.72 a	20.28 e	5.55 e	6.66 c	19.09 a	74.08 a
		T2	59.84 d	42.58 b	13.30 b	7.49 ab	18.26 bcd	71.42 cd
		T3	50.26 e	54.64 a	21.00 a	7.94 a	17.74 d	70.42 d
	S2	T1	63.72 a	20.28 e	5.55 e	6.66 c	19.09 a	74.08 a
		T2	60.68 cd	33.81 cd	10.70 bc	7.75 ab	18.16 bcd	73.00 abc
		T3	59.96 d	38.51 bc	11.15 bc	7.86 ab	17.91 cd	71.92 bcd
	S3	T1	63.72 a	20.28 e	5.55 e	6.66 c	19.09 a	74.08 a
		T2	63.06 ab	20.94 e	6.86 de	7.26 bc	18.68 ab	73.75 a
		T3	61.86 bc	30.75 d	9.35 cd	7.31 abc	18.53 abc	73.50 ab
V2	S1	T1	66.34 a	14.64 e	4.60 d	6.79 d	25.48 a	75.92 a
		T2	60.99 cd	34.15 ab	9.38 b	7.43 bc	24.00 bc	73.83 c
		T3	56.42 e	38.18 a	12.21 a	8.15 a	23.24 c	72.34 d
	S2	T1	66.34 a	14.64 e	4.60 d	6.79 d	25.48 a	75.92 a
		T2	61.91 c	22.53 d	8.58 bc	7.68 abc	23.80 bc	74.50 bc
		T3	58.88 d	32.87 b	9.33 b	7.92 ab	23.60 bc	73.67 c
	S3	T1	66.34 a	14.64 e	4.60 d	6.79 d	25.48 a	75.92 a
		T2	64.71 ab	18.06 e	5.52 d	7.33 c	24.44 b	75.38 ab
		T3	62.18 bc	26.61 c	7.71 c	7.59 bc	24.32 b	74.50 bc
ANOVA		V	***	***	**	ns	***	***
		S	***	***	***	*	**	***
		T	***	***	***	***	***	***
		V*S	**	*	**	ns	ns	ns
		V*T	ns	ns	**	ns	*	ns
		S*T	***	***	***	ns	ns	*
		V*S*T	**	ns	*	ns	ns	ns

S1 represents irrigating saline from the regreening stage to the panicle initiation stage; S2 represents irrigating saline from the panicle initiation stage to the flowering stage; S3 represents irrigating saline from the flowering stage to the maturity stage. T1, T2 and T3 represent the 0‰ saline, 3‰ saline and 6‰ saline, respectively. V1 and V2 represent the varieties of CY1000 and LLY506, respectively. Different lower-case letters of the same variety represent significant differences at 0.05 probability level according to LSD. *** represents the significant difference at the 0.001 level according to the LSD test, ** represents the significant difference at the 0.01 level according to the LSD test, * represents the significant difference at the 0.05 level according to the LSD test, and ns represents no significant difference.

**Table 6:** Effect of irrigated saline water and different saline concentrations at different growth stages on grain quality (2022).

Variety	Irrigation period	Salinity	Head rice (%)	Chalky grain (%)	Chalkiness (%)	Protein content (%)	Amylose content (%)	Taste value
V1	S1	T1	64.05 a	18.97 f	6.91 f	6.46 e	19.14 a	76.63 a
		T2	56.82 de	33.24 d	12.82 c	7.21 c	18.16 bc	74.25 bc
		T3	52.81 f	45.96 b	17.83 b	8.09 b	17.35 e	73.13 c
	S2	T1	64.05 a	18.97 f	6.91 f	6.46 e	19.14 a	76.63 a
		T2	60.76 bc	25.08 e	9.71 de	7.23 c	18.44 b	75.38 ab
		T3	58.29 cd	36.21 c	13.29 c	7.83 b	17.54 de	73.63 c
	S3	T1	64.05 a	18.97 f	6.91 f	6.46 e	19.14 a	76.63 a
		T2	62.38 ab	21.79 f	8.40 ef	6.74 de	18.50 b	75.63 ab
		T3	60.31 bc	27.91 e	10.26 d	7.04 cd	17.86 cd	74.50 bc
	S4	T1	64.05 a	18.97 f	6.91 f	6.46 e	19.14 a	76.63 a
		T2	54.14 ef	38.38 c	14.40 c	7.69 b	17.38 e	73.38 c
		T3	44.22 g	61.68 a	25.10 a	8.61 a	16.26 f	69.63 d
V2	S1	T1	63.95 a	20.63 g	6.79 f	6.58 d	24.23 a	76.75 a
		T2	57.17 cd	34.47 d	11.53 c	7.35 c	23.08 cd	74.50 cde
		T3	51.68 e	42.72 b	14.39 b	7.95 b	22.78 de	73.38 e
	S2	T1	63.95 a	20.63 g	6.79 f	6.58 d	24.23 a	76.75 a
		T2	60.33 bc	31.67 de	8.63 de	7.51 c	23.25 cd	75.38 bc
		T3	53.79 de	37.92 c	9.87 d	7.97 b	23.09 cd	74.25 cde
	S3	T1	63.95 a	20.63 g	6.79 f	6.58 d	24.23 a	76.75 a
		T2	61.76 ab	24.64 f	8.03 ef	6.91 d	23.89 ab	76.50 ab
		T3	57.61 c	30.89 e	8.71 de	7.55 c	23.43 bc	74.75 cd
	S4	T1	63.95 a	20.63 g	6.79 f	6.58 d	24.23 a	76.75 a
		T2	53.40 e	42.51 b	12.87 bc	8.01 b	22.34 e	73.50 de
		T3	35.48 f	55.41 a	17.37 a	9.16 a	20.05 f	70.88 f
ANOVA		V	ns	ns	**	ns	***	ns
		S	***	***	***	***	***	***
		T	***	***	***	***	***	***
		V*S	ns	**	***	ns	**	ns
		V*T	***	***	***	ns	ns	ns
		S*T	***	***	***	***	***	***
		V*S*T	ns	*	*	ns	**	ns

S1 represents irrigating saline from the regreening stage to the panicle initiation stage; S2 represents irrigating saline from the panicle initiation stage to the flowering stage; S3 represents irrigating saline from the flowering stage to the maturity stage; S4 represents irrigating saline from the regreening stage to the maturity stage. T1, T2 and T3 represent the 0‰ saline, 3‰ saline and 6‰ saline, respectively. V1 and V2 represent the varieties of CY1000 and LLY506, respectively. Different lower-case letters at the same variety represent significant different at 0.05 probability level according to LSD. *** represents the significant difference at the 0.001 level according to LSD test, ** represents the significant difference at the 0.01 level according to LSD test, * represents the significant difference at the 0.05 level according to LSD test, ns represents no significant difference.

### 3.5 Effects of irrigating brine at different growth periods with various saline concentrations on the content of Na^+^ and K^+^ in the shoot

**Figure 4:**
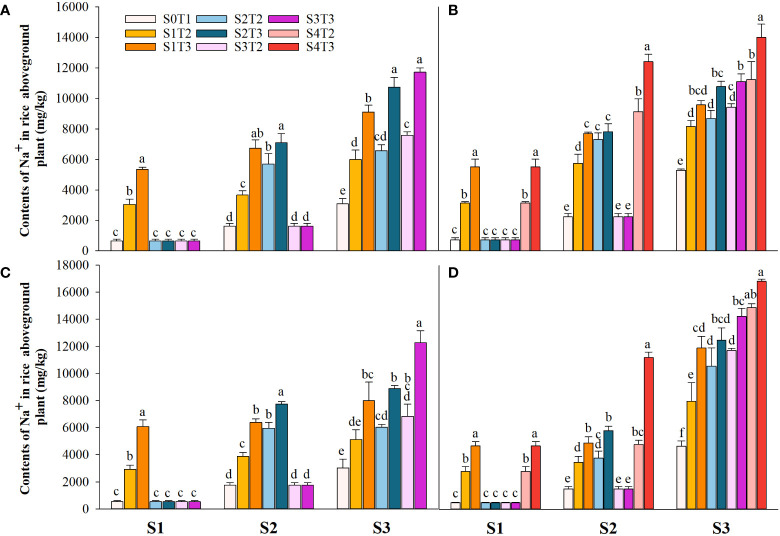
Effects of irrigating brine at different growth stages with different brine concentrations on aboveground Na^+^ content of CY1000 **(A, B)** and LLY506 **(C, D)** in 2021 **(A, C)** and in 2022 **(B, D)**. Error bars are ± SE. S0T1 (CK) represents irrigating with 0‰ brine during the whole growth stage; S1T2 represents irrigating 3‰ brine from the regreening stage to the panicle initiation stage; S1T3 represents irrigating 6‰ brine from the regreening stage to the panicle initiation stage; S2T2 represents irrigating 3‰ brine from the panicle initiation stage to the flowering stage; S2T3 represents irrigating 6‰ brine from the panicle initiation stage to the flowering stage; S3T2 represents irrigating 3‰ brine from the flowering stage to the maturity stage; S3T3 represents irrigating 6‰ brine from the flowering stage to the maturity stage; S4T2 represents irrigating 3‰ brine from the regreening stage to the maturity stage; S4T3 represents irrigating 6‰ brine from the regreening stage to the maturity stage; S1, S2 and S3 represent the panicle initiation stage, the flowering stage and the maturity stage, respectively. Different lowercase letters at the same growth stage indicate significant differences at 0.05 levels, according to the LSD test.

**Figure 5:**
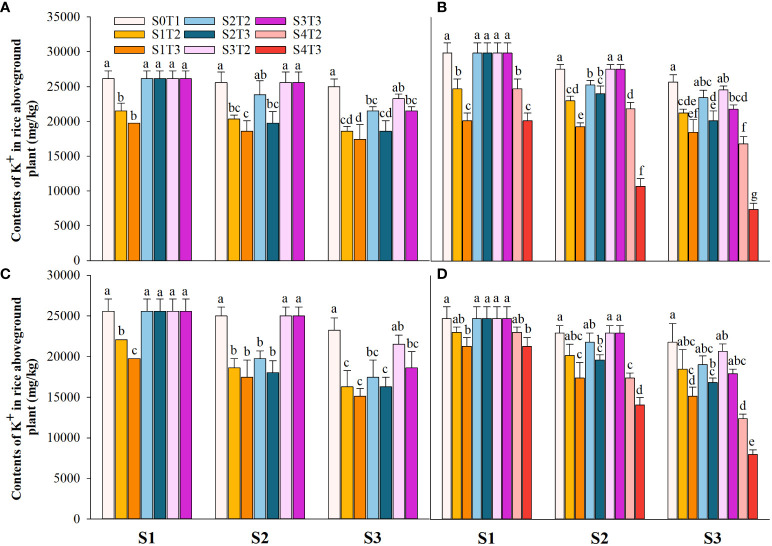
Effects of irrigating brine at different growth stages with different brine concentrations on aboveground K^+^ content of CY1000 **(A, B)** and LLY506 **(C, D)** in 2021 **(A, C)** and in 2022 **(B, D)**. Error bars are ± SE. S0T1 (CK) represents irrigating with 0‰ brine during the whole growth stage; S1T2 represents irrigating 3‰ brine from the regreening stage to the panicle initiation stage; S1T3 represents irrigating 6‰ brine from the regreening stage to the panicle initiation stage; S2T2 represents irrigating 3‰ brine from the panicle initiation stage to the flowering stage; S2T3 represents irrigating 6‰ brine from the panicle initiation stage to the flowering stage; S3T2 represents irrigating 3‰ brine from the flowering stage to the maturity stage; S3T3 represents irrigating 6‰ brine from the flowering stage to the maturity stage; S4T2 represents irrigating 3‰ brine from the regreening stage to the maturity stage; S4T3 represents irrigating 6‰ brine from the regreening stage to the maturity stage; S1, S2 and S3 represent the panicle initiation stage, the flowering stage and the maturity stage, respectively. Different lowercase letters at the same growth stage indicate significant differences at 0.05 levels, according to the LSD test.

**Figure 6:**
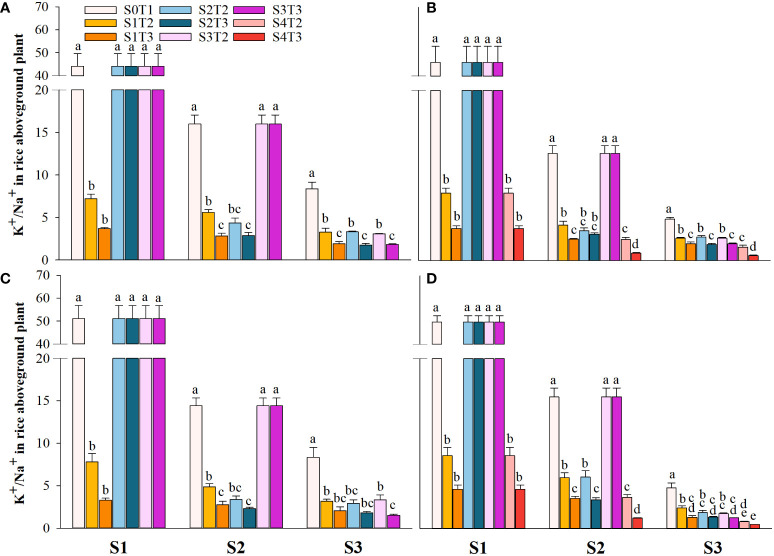
Effects of irrigating brine at different growth stages with different brine concentrations on aboveground K^+^/Na^+^ of CY1000 **(A, B)** and LLY506 **(C, D)** in 2021 **(A, C)** and in 2022 **(B, D)**. Error bars are ± SE. S0T1 (CK) represents irrigating with 0‰ brine during the whole growth stage; S1T2 represents irrigating 3‰ brine from the regreening stage to the panicle initiation stage; S1T3 represents irrigating 6‰ brine from the regreening stage to the panicle initiation stage; S2T2 represents irrigating 3‰ brine from the panicle initiation stage to the flowering stage; S2T3 represents irrigating 6‰ brine from the panicle initiation stage to the flowering stage; S3T2 represents irrigating 3‰ brine from the flowering stage to the maturity stage; S3T3 represents irrigating 6‰ brine from the flowering stage to the maturity stage; S4T2 represents irrigating 3‰ brine from the regreening stage to the maturity stage; S4T3 represents irrigating 6‰ brine from the regreening stage to the maturity stage; S1, S2 and S3 represent the panicle initiation stage, the flowering stage and the maturity stage, respectively. Different lowercase letters at the same growth stage indicate significant differences at 0.05 levels, according to the LSD test. The break-up ranged from 20 to 40 centimeters (**Figure 6**).

Saline irrigation at different growth periods and concentrations significantly affected aboveground Na^+^ and K^+^ contents (mg kg^-1^) and K^+^/Na^+^ in rice at all periods. **Figures 4**–**6** show that the aboveground Na^+^ content increased rapidly while K^+^ and K^+^/Na^+^ decreased when the two rice varieties were subjected to saline stress at different periods. When the rice was saline stressed at the S1, the aboveground Na^+^ content increased 375.5%−733.3%, 455.1%−1053.0% and 338.8%−670.0%, 461.7%−838.1% in CY1000 and LLY506 in 2021 and 2022, respectively. The aboveground K^+^ content was reduced by 17.8%−24.5%, 13.6%−22.7%, and 17.2%−32.5%, 6.9%−13.9%, respectively; K^+^/Na^+^ was reduced by 83.7%−91.6%, 84.7%−93.5% and 82.8%−91.9%, 82.8%−90.7%, respectively, which shows that the differences among treatments with increasing saline concentration were highly significant at S1. The changes in the magnitude of Na^+^ content in the aboveground of the two varieties of rice at the flowering stage were S2 > S1 > CK, and K^+^ content and K^+^/Na^+^ were CK > S1 > S2. At the maturity stage of rice, the changes in the magnitude of Na^+^ content were S3 > S2 >S1 > CK, and the changes in the magnitude of K^+^ content were CK > S1 > S2 > S3, while the differences in K^+^/Na^+^ under S1, S2 and S3 treatments at the same salinity were small.

## 4 Discussion

### 4.1 Rice growth sensitivity to salinity at different growth stages

In different growth stages of rice, irrigation with saline water resulted in decreased plant height, tillering number, and aboveground biomass. The inhibition effect of saline water was more significant with the increased saline water concentration (**Figures 1**–**3**). The adverse effects of salt stress on rice growth and development were mainly attributed to decreased water potential in the short term and the toxicity caused by long-term Na^+^ accumulation (Yan et al., 2020). High salt stress seriously affected rice cell division and cell elongation, resulting in impaired root establishment, leaf rolling, loss of green, decreased tillering number per plant, decreased biomass, and shorter plant height (Munns, 2002; Hakim et al., 2014; Van Zelm et al., 2020). It is reported that the vegetative growth stage before PI is the most active period for the initiation of rice tillers, which determines the stem weight of rice and the number of tillers per plant (Zeng et al., 2001). This is consistent with the results of this study. Compared with S2 and S3, the reduction of plant height, tiller number, and aboveground rice biomass under irrigation brine in S1 were more severe. Salt stress from regreening to panicle initiation stage of rice leads to the stunted growth of rice, reduces nutrients uptake, inhibits the allocation of biomass, and obstructs the transport of photoassimilates to other organs (Dramalis et al., 2020), significantly reducing the development of plant height, tillers and leaf surface area of rice (Ashrafuzzaman et al., 2003; Shakri et al., 2022).

### 4.2 Rice yield response in different growth stages under salinity

Irrigation with brine significantly reduced grain yield at all growth stages of rice, and the yield penalty was more severe with the increase of irrigation brine concentration (**Tables 1**, **2**). Rice yield results from synthesizing yield factors determined by photosynthetic substances production capacity and the assimilate allocation (Zhou et al., 2018). Previous studies showed that salt stress inhibited rice growth and development and significantly affected rice yield traits such as seed setting rate, tiller number, panicle number, and panicle length, among which stunted spikelets, especially inferior spikelets, significantly reduced rice grain yield (Fu et al., 2011; Zhang et al., 2015); Shereen et al. (2005) reported that seed setting rate was the main cause of yield loss under brine condition. Furthermore, the decrease in tillering number per plant and grain number per panicle is the main reason for rice yield loss under salt stress (Zeng and Shannon, 2000; Dolferus et al., 2011). The results of our study revealed that the grain yield decline of two rice varieties in different growth stages under saline stress was S1 > S2 > S3. Moreover, we found that the rice yield is more dependent on the exposure of rice to salinity at the S1 stage, as evidenced by the fact that when the rice was irrigated with saline water only one time at the S1 stage, later, even though irrigation with freshwater couldn’t recover the growth development of rice. Irrigation with brine at the S1 stage posed a yield penalty of 63.6%, which was 51.8% and 233.0% higher than the S2 and S3 growth stages (**Tables 1**, **2**). This indicates that the compensation effect of salt stress on the yield is highly dependent on the reduction of the yield components in the early period, such as the number of tillers and the number of grains per panicle (**Tables 1**, **2**). Saline water stress in the early stage of young spike differentiation can lead to the degradation of spikelets primordium, affect the germination of spikelets and the formation of spikelets, lead to the increase of the number of sterile florets and decrease the number of spikelets in the panicle (Khatun and Flowers, 1995; Abdullah et al., 2001; Hasanuzzaman et al., 2009). However, sterility of rice spikelets in the rice reproductive stage is considered the main threat under salt stress (Yeo and Flowers, 1984). In this experiment, spikelet degradation was caused by irrigation brine from the differentiation stage of young panicles to the flowering stage, significantly reducing the number of grains per unit area and yield. The application of salt after the flowering stage impeded the transport of photosynthates to the filling site (Abdullah et al., 2001), reducing the accumulation of dry matter, the filling rate, and the filling rate and resulted in the decrease of the 1000-grain weight of rice. Still, the sensitivity of the tiller number to the salt applied after the flowering stage was small (Zeng et al., 2001). Therefore, irrigation with saline water after the flowering period has less yield loss than other periods. Based on comprehensive analysis, it is consistent with Ebrahimi et al. (2012) that saline irrigation at the early growth stage has more negative effects on yield and its component factors, and the decrease of panicle number per unit area and kernel number per panicle is the leading factor of yield decrease under salt stress. Therefore, in the whole growth process of rice production in saline areas, more attention should be paid to avoiding the interference of salt during the regreening stage and the young panicle differentiation stage of rice to reduce the influence of salt stress on grain yield.

### 4.3 The mechanism behind the rice grain quality deterioration

Irrigation with brine at different growth stages of rice resulted in decreased rice quality, and the S1 stage was more sensitive to salt (**Tables 5**, **6**). It was found that the production capacity of photosynthetic substances and the distribution of assimilate function affected the quality of rice. At the same time, salt stress limits the absorption and utilization of rice nutrients, resulting in nutrient deficiency or imbalance. Grain filling is a dynamic process related to sourcing-sink balance, in which the source is the cornerstone for the formation of rice quality. Grain-filling substances are mainly derived from the storage materials of stem sheaths before heading and the photosynthates after heading (Ishimaru et al., 2020; Liu et al., 2022). Therefore, the size of crop nutrients, which are accumulated and used as photosynthetic products during photosynthesis, determines crop final yield and quality. In this study, it was found that the rapid accumulation of Na^+^ in rice under salt stress resulted in the imbalance of intracellular ions, limited the absorption of K^+^ necessary for maintaining normal physiological activities of the crop, and significantly reduced K^+^/Na^+^ (**Figures 4**–**6**). It has been reported that a large accumulation of Na^+^ disrupts the structure and function of nitrogen metabolizing enzymes and limits the ability of rice to reduce and assimilate N (He and Zhu, 2008; Hossain et al., 2012; Ashraf et al., 2018). At the same time, after leaf stomata were closed under osmotic stress, the intercellular CO_2_ concentration decreased (Oi et al., 2019), the photosynthetic rate decreased significantly, and the process of photosynthate transport to the grain was inhibited (Razzaq et al., 2020). In this study, it was found that in the same brine treatment for the same time, the K^+^/Na^+^ in rice was still significantly lower than that in the control until harvest after S1 was irrigated with fresh water after salt stress. The aboveground nitrogen uptake and IE_N_ were the lowest after S1 treatment and inversely proportional to irrigated brine concentration. According to the experimental results, it is speculated that the vegetative growth stage before PI is the period when rice tillers are robust, and the source-sink relationship is rapidly established. During this period, irrigation brine significantly inhibits the absorption and utilization of nitrogen, the accumulation of carbohydrates in the early stage of grain filling, and the development of photosynthetic organs after the heading is blocked. The stunted growth of rice nutrients leads to the weakened source-sink relationship and limits the rice filling process degrading rice quality (Won et al., 2022). The in-compaction of endosperm tissue leads to decreased starch accumulation, increased chalkiness grain rate, and chalkiness area (Fahad et al., 2019), and easy breakage in the processing process and milling leads to the low yield of whole rice. The increased Na^+^ concentration inhibits starch synthase activity, and the amylose content in grains significantly decreases (Rao et al., 2013) and substantially increases grain storage protein content (Wani et al., 2012; Kumar and Khare, 2016), affecting the cooking characteristics of rice, reducing the edible quality and restricting the marketing circulation (Chun et al., 2009; Sawada et al., 2016; Ayaad et al., 2021). In this study, compared with S2 and S3, the salt stress in the S1 stage resulted in a lower grain finishing rice rate, increased chalkiness and protein content, and decreased amylase content and taste value. This verified that salt stress before PI negatively affected rice filling more than other growth periods.

In summary, irrigation with brine at the S1 stage reduced the nitrogen uptake, and IE_N_, which resulted in rice yield penalty and deteriorated rice quality. On the other hand, even though the Na^+^ content in the S2 and S3 plants was higher, the rice quality was still higher than in the S1 plants (with lower Na^+^ content). This further shows that at the S1 stage, salinity reduces the nitrogen uptake, resulting in stunted plant growth, reducing tillering, yield, and yield components, and deteriorating the rice quality. Therefore, it is a good way to avoid yield penalty and deterioration of rice quality when planting rice in salt-affected areas and ensuring fresh water irrigation during S1 stage.

### 4.4 Consideration of the application of saline irrigation according to the sensitivity of different growth stages of rice

Irrigated agriculture is the leading consumer of global water resources, accounting for nearly 70% of the global total water withdrawal (United Nations Educational & Organization, 2020). The available freshwater resources in coastal saline areas are more limited. The results showed that the loss of rice yield and quality due to long-term salt stress was unacceptable. However, there are differences in the sensitivity of rice to salt at different growth stages. By determining the salt-sensitive or salt-tolerant growth period of rice, and formulating management plans, sufficient seawater resources in coastal areas could be rationally utilized without significantly affecting the yield and quality of rice, and field irrigation management measures can be formulated according to local conditions, to improve the loss caused by salt and save precious fresh water resources at the same time (Zeng et al., 2001; Wang et al., 2007; Vergine et al., 2017).

Moreover, the negative effect of S4 irrigation brine on rice is highly significant, and the loss of rice production is enormous. While in the S1 period, irrigation brine had a more substantial loss in rice yield and quality than S2 and S3, and the loss of irrigation brine was the least after the flowering period. Therefore, S1 was the critical period of rice salt sensitivity. When planting rice in saline areas, it is necessary to ensure the quality of fresh water for rice irrigation in the S1 period as much as possible. After the flowering period, partial irrigation replacement with light saline water can maintain grain yield stability and quality. This irrigation strategy provides a management option for using or reusing saltwater in many agricultural areas. If the influence of crop type and salt treatment timing can be combined with the threshold value of salt effect on yield and quality, irrigation practices, soil texture, meteorological information, and other factors can be developed to mitigate further the impact of salt in crop production on crop yield and quality decline. Soil tillage, agricultural production, and saline water application will be better developed. There is massive potential in the future, which is worth exploring in depth.

Furthermore, previous studies have also done some work on salt stress in rice. Zheng et al. (2023) believed that rice grain yield was significantly reduced, while the effect of salinity initiated in the reproductive stage was more significant than that started in the seedling stage. Osmotic and ionic stress seriously inhibited the reproductive process of rice (pollination and fertilization), and further resulted in the abortion or degeneration of spikelet and the decrease of filled grains rate (Shereen et al., 2005; Chen et al., 2021). Compared with continuous irrigation of fresh water, the yield of rice irrigated with 3 ‰ salt water at different growth stages decreased by 44.1% at booting stage, 39.0% at jointing stage, 22.5% at heading stage and 6.8% at filling stage. The study found that the decrease of seed setting rate was the biggest reason for the loss of rice yield caused by salt stress at booting stage (Zhu and Fan, 2021). Nonetheless, the results of the previous were documented from well-controlled pot experiments. However, the findings of our research suggested that rice was more sensitive to salt stress from the regreening to panicle initiation stage, which was inconsistent with the previously reported research work. The present research was carried out on a coastal open field. In addition, the base has advanced pipeline facilities, which could directly pump seawater and underground fresh water to be blended into the required concentration of salt water to transport to each experimental area. Based on the field experiment, the nitrogen content of rice, salt ions in the plants and various grain qualities were also determined, and it was found that during the regreening to the panicle initiation stage, irrigation brine inhibited the nitrogen uptake of rice and caused great damage to rice. Even after this period, replacement of freshwater irrigation until maturity stage could not compensate for the previous losses of salt, and ultimately significantly inhibited the formation of yield and quality. Therefore, the present research concluded that under the open field conditions rice was more sensitive to salt stress from the regreening to panicle initiation stage (S1) while reproductive stage (S2 or S3) was comparatively less sensitive to salt stress.

## 5 Conclusion

Salinity mainly reduced the nitrogen uptake, resulting in stunted plant growth, reducing tillering, yield, and yield components, and deteriorating the rice quality. The rice varieties were most sensitive to irrigating brine treatment during S1 under a similar processing time. The grain yield and quality decreased as the brine concentration was increased. When planting rice in saline areas, it is necessary to ensure the quality of fresh water for rice irrigation in the S1 period as much as possible. After the flowering period, low salt water can be irrigated appropriately, which could still maintain stable grain yield and quality to a certain extent. However, the underlying mechanism of salt stress on different types of rice in different growth periods is unclear and needs to be addressed further.

## Data availability statement

The raw data supporting the conclusions of this article will be made available by the authors, without undue reservation.

## Author contributions

YL, LN, ZA and MK planned and designed the research. YL, YM, TZ, YZ, LL and ZH performed the experiments. YL wrote the manuscript. LN, ZA and MK supervised this work. LN, ZA and MK reviewed the manuscript. All authors contributed to the article and approved the submitted version. All authors approved the manuscript for publication.
