# Supplementary material for: Rice yield penalty and quality deterioration is associated with failure of nitrogen uptake from regreening to panicle initiation stage under salinity
Source: Front Plant Sci. 2023 Mar 21;14:1120755. doi: 10.3389/fpls.2023.1120755 (PMC10071828; doi:10.3389/fpls.2023.1120755)
Supplement: Supplementary file 1 [file Table_1.docx]

**SUPPLEMENTARY TABLE 1** Effects of irrigated saline water and different saline concentrations at different growth stages on rice yield components and quality (2021)

| Variety | Irrigation period | Salinity | Spikelets  (m^-2^×10^3^) | Biomass  (t ha^-1^) | Harvest  index | Brown  rice (%) | Milled rice (%) |  |
| --- | --- | --- | --- | --- | --- | --- | --- | --- |
| V1 | S1 | T1 | 43.2 a | 21.2 a | 0.43 a | 80.69 a | 68.67 a |  |
|  |  | T2 | 24.4 f | 15.2 e | 0.27 e | 79.48 b | 67.06 b |  |
|  |  | T3 | 15.6 g | 13.8 g | 0.17 f | 76.39 c | 61.16 c |  |
|  | S2 | T1 | 43.2 a | 21.2 a | 0.43 a | 80.69 a | 68.67 a |  |
|  |  | T2 | 34.4 d | 15.7 d | 0.38 b | 80.34 ab | 67.82 ab |  |
|  |  | T3 | 30.4 e | 14.5 f | 0.32 d | 80.01 ab | 67.61 ab |  |
|  | S3 | T1 | 43.2 a | 21.2 a | 0.43 a | 80.69 a | 68.67 a |  |
|  |  | T2 | 39.7 b | 18.5 b | 0.39 b | 80.60 a | 68.43 ab |  |
|  |  | T3 | 37.2 c | 17.7 c | 0.36 c | 80.35 ab | 67.96 ab |  |
| V2 | S1 | T1 | 53.6 a | 23.9 a | 0.44 a | 80.37 a | 69.88 a |  |
|  |  | T2 | 27.8 f | 15.8 e | 0.26 f | 78.20 bc | 65.88 cd |  |
|  |  | T3 | 21.4 g | 13.4 g | 0.22 g | 75.46 d | 62.75 e |  |
|  | S2 | T1 | 53.6 a | 23.9 a | 0.44 a | 80.37 a | 69.88 a |  |
|  |  | T2 | 41.0 d | 16.9 d | 0.37 c | 78.87 ab | 66.93 bc |  |
|  |  | T3 | 33.0 e | 14.6 f | 0.31 e | 77.21 c | 64.23 de |  |
|  | S3 | T1 | 53.6 a | 23.9 a | 0.44 a | 80.37 a | 69.88 a |  |
|  |  | T2 | 47.4 b | 20.1 b | 0.39 b | 79.68 ab | 68.17 ab |  |
|  |  | T3 | 43.8 c | 19.1 c | 0.35 d | 78.97 ab | 66.94 bc |  |
| ANOVA | | | V | *** | ** | ns | * | ns |
|  |  |  | S | *** | *** | *** | *** | *** |
|  |  |  | T | *** | *** | *** | *** | *** |
|  |  |  | V*S | * | *** | ** | ns | * |
|  |  |  | V*T | *** | *** | ** | * | ** |
|  |  |  | S*T | *** | *** | *** | *** | *** |
|  |  |  | V*S*T | *** | ns | *** | ns | ** |

**Note:** S1 represents irrigating saline from the regreening stage to the panicle initiation stage; S2 represents irrigating saline from the panicle initiation stage to the flowering stage; S3 represents irrigating saline from the flowering stage to the maturity stage. T1, T2 and T3 represent the 0‰ saline, 3‰ saline and 6‰ saline, respectively. V1 and V2 represent the varieties of CY1000 and LLY506, respectively. Different lower-case letters of the same variety represent significant differences at 0.05 probability level according to LSD. *** represents the significant difference at the 0.001 level according to the LSD test, ** represents the significant difference at the 0.01 level according to the LSD test, * represents the significant difference at the 0.05 level according to the LSD test, and ns represents no significant difference.

**SUPPLEMENTARY TABLE 2** Effects of irrigated saline water and different saline concentrations at different growth stages on rice yield components and quality (2022)

| Variety | Irrigation period | Salinity | Spikelets  (m^-2^×10^3^) | Biomass  (t ha^-1^) | Harvest  index | Brown  rice (%) | Milled rice (%) |  |
| --- | --- | --- | --- | --- | --- | --- | --- | --- |
| V1 | S1 | T1 | 45.9 a | 25.8 a | 0.46 a | 81.05 a | 68.73 a |  |
|  |  | T2 | 23.3 f | 14.7 f | 0.34 d | 79.97 bc | 67.74 b |  |
|  |  | T3 | 16.4 g | 12.5 g | 0.26 f | 78.67 d | 65.49 c |  |
|  | S2 | T1 | 45.9 a | 25.8 a | 0.46 a | 81.05 a | 68.73 a |  |
|  |  | T2 | 32.9 d | 19.2 d | 0.38 c | 80.50 ab | 68.23 ab |  |
|  |  | T3 | 27.5 e | 15.8 e | 0.34 d | 80.27 b | 67.88 b |  |
|  | S3 | T1 | 45.9 a | 25.8 a | 0.46 a | 81.05 a | 68.73 a |  |
|  |  | T2 | 40.3 b | 21.9 b | 0.42 b | 81.01 a | 68.30 ab |  |
|  |  | T3 | 36.9 c | 20.4 c | 0.37 c | 80.91 a | 68.14 ab |  |
|  | S4 | T1 | 45.9 a | 25.8 a | 0.46 a | 81.05 a | 68.73 a |  |
|  |  | T2 | 21.3 f | 12.9 g | 0.31 e | 79.58 c | 65.87 c |  |
|  |  | T3 | 8.4 h | 6.2 h | 0.14 g | 77.28 e | 59.69 d |  |
| V2 | S1 | T1 | 52.9 a | 22.1 a | 0.46 a | 79.19 a | 69.33 a |  |
|  |  | T2 | 27.2 e | 13.3 d | 0.32 d | 75.97 abc | 65.74 bc |  |
|  |  | T3 | 19.7 f | 10.2 e | 0.27 e | 74.01 c | 63.85 c |  |
|  | S2 | T1 | 52.9 a | 22.1 a | 0.46 a | 79.19 a | 69.33 a |  |
|  |  | T2 | 36.6 d | 15.3 c | 0.39 c | 76.41 abc | 66.35 abc |  |
|  |  | T3 | 28.8 e | 11.5 e | 0.33 d | 75.51 bc | 64.90 bc |  |
|  | S3 | T1 | 52.9 a | 22.1 a | 0.46 a | 79.19 a | 69.33 a |  |
|  |  | T2 | 47.7 b | 18.4 b | 0.43 b | 78.04 ab | 67.64 ab |  |
|  |  | T3 | 42.9 c | 16.5 c | 0.39 c | 77.79 ab | 66.40 abc |  |
|  | S4 | T1 | 52.9 a | 22.1 a | 0.46 a | 79.19 a | 69.33 a |  |
|  |  | T2 | 20.6 f | 11.0 e | 0.26 e | 75.30 bc | 64.82 bc |  |
|  |  | T3 | 8.1 g | 6.13 f | 0.11 f | 60.51 d | 47.50 d |  |
| ANOVA | | | V | ** | *** | ns | *** | * |
|  |  |  | S | *** | *** | *** | *** | *** |
|  |  |  | T | *** | *** | *** | *** | *** |
|  |  |  | V*S | *** | *** | ** | *** | ** |
|  |  |  | V*T | *** | * | ns | *** | *** |
|  |  |  | S*T | *** | *** | *** | *** | *** |
|  |  |  | V*S*T | * | * | ns | *** | *** |

**Note:** S1 represents irrigating saline from the regreening stage to the panicle initiation stage; S2 represents irrigating saline from the panicle initiation stage to the flowering stage; S3 represents irrigating saline from the flowering stage to the maturity stage; S4 represents irrigating saline from the regreening stage to the maturity stage. T1, T2 and T3 represent the 0‰ saline, 3‰ saline and 6‰ saline, respectively. V1 and V2 represent the varieties of CY1000 and LLY506, respectively. Different lower-case letters of the same variety represent significant differences at 0.05 probability level according to LSD. *** represents the significant difference at the 0.001 level according to the LSD test, ** represents the significant difference at the 0.01 level according to the LSD test, * represents the significant difference at the 0.05 level according to the LSD test, and ns represents no significant difference.
